# Comparative genome analysis of the genus *Shewanella* unravels the association of key genetic traits with known and potential pathogenic lineages

**DOI:** 10.3389/fmicb.2023.1124225

**Published:** 2023-02-28

**Authors:** Gabriela N. Cerbino, German M. Traglia, Teolincacihuatl Ayala Nuñez, Gisela Parmeciano Di Noto, María Soledad Ramírez, Daniela Centrón, Andrés Iriarte, Cecilia Quiroga

**Affiliations:** ^1^Universidad de Buenos Aires, Consejo Nacional de Investigaciones Científicas y Tecnológicas, Instituto de Investigaciones en Microbiología y Parasitología Médica (IMPAM), Facultad de Medicina, Buenos Aires, Argentina; ^2^Laboratorio de Biología Computacional, Departamento de Desarrollo Biotecnológico, Instituto de Higiene, Facultad de Medicina, Universidad de la República, Montevideo, Uruguay; ^3^Center for Applied Biotechnology Studies, Department of Biological Science, California State University, Fullerton, Fullerton, CA, United States

**Keywords:** *Shewanella*, accessory genome, mobilome, resistome, virulome, horizontal gene transfer

## Abstract

*Shewanella* spp. are Gram-negative rods widely disseminated in aquatic niches that can also be found in human-associated environments. In recent years, reports of infections caused by these bacteria have increased significantly. Mobilome and resistome analysis of a few species showed that they are versatile; however, comprehensive comparative studies in the genus are lacking. Here, we analyzed the genetic traits of 144 genomes from *Shewanella* spp. isolates focusing on the mobilome, resistome, and virulome to establish their evolutionary relationship and detect unique features based on their genome content and habitat. *Shewanella* spp. showed a great diversity of mobile genetic elements (MGEs), most of them associated with monophyletic lineages of clinical isolates. Furthermore, 79/144 genomes encoded at least one antimicrobial resistant gene with their highest occurrence in clinical-related lineages. CRISPR-Cas systems, which confer immunity against MGEs, were found in 41 genomes being I-E and I-F the more frequent ones. Virulome analysis showed that all *Shewanella* spp. encoded different virulence genes (motility, quorum sensing, biofilm, adherence, etc.) that may confer adaptive advantages for survival against hosts. Our data revealed that key accessory genes are frequently found in two major clinical-related groups, which encompass the opportunistic pathogens *Shewanella algae* and *Shewanella xiamenensis* together with several other species. This work highlights the evolutionary nature of *Shewanella* spp. genomes, capable of acquiring different key genetic traits that contribute to their adaptation to different niches and facilitate the emergence of more resistant and virulent isolates that impact directly on human and animal health.

## 1. Introduction

In recent years, we have observed an increase in reports of infections caused by the pathogen *Shewanella* (Ng et al., 2022). *Shewanella* spp. are Gram-negative rods, widely disseminated in aquatic niches (mainly marine environments), sediments and soil, that can cause infections in humans. Most infections are attributed to a few *Shewanella* species that may cause skin and soft tissue infections, bacteriemia, hepatobiliary infections, otitis media, etc. (Sharma and Kalawat, 2010; Yousfi et al., 2017; Ng et al., 2022). Although most infection-related species were identified as *Shewanella algae* and *Shewanella putrefaciens*, in the last years the employment of molecular based-methods improved the identification of other pathogenic species, such as *S. xiamenensis* (Parmeciano Di Noto et al., 2016; Thorell et al., 2019; Huang et al., 2022). Furthermore, some *Shewanella* species can also cause infections in aquatic animals (e.g., *S. algae*) or coexist as symbionts or epibionts (e.g., *Shewanella pealeana* and *Shewanella woodyi*) (Hau and Gralnick, 2007; Janda and Abbott, 2014); while other species have shown to be important dissimilatory metal-reducing bacteria (Zhong et al., 2018). In order to thrive in each environment *Shewanella* spp. encode a plethora of genes that may contribute to their diversification and niche adaptation.

Previous studies with few isolates showed that this genus is capable of acquiring a wide variety of mobile elements, such as plasmids, prophages, group II (GII) introns, integrons, and integrative and conjugative elements (ICEs) (Pembroke and Piterina, 2006; Larouche and Roy, 2009; Quiroga and Centrón, 2009; Ramírez et al., 2010; Carattoli, 2013; Parmeciano Di Noto et al., 2016; Fang et al., 2018; Parmeciano Di Noto et al., 2019; Thorell et al., 2019; Huang et al., 2022). Recently, a comprehensive analysis of integron integrases reported that multiple horizontal genetic transfer (HGT) events were implicated in the emergence and spread of novel mobile integrons (Nuñez et al., 2022). Genome analyses have also led to the identification of a few resistance plasmids in *Shewanella* spp. isolates that participate in antimicrobial resistance (AMR) dissemination (Carattoli, 2013; Zago et al., 2020).

In the last decade *Shewanella* spp. have become more relevant due to its role as reservoir of AMR genes *bla*_OXA–48_ and *qnrA*, from which were most likely transferred to *Enterobacteriaceae* and other frequent human pathogens (Dabos et al., 2018; Tacão et al., 2018; Araújo et al., 2021). In addition, there has been an increase in reports on multidrug resistant (MDR) *Shewanella* spp. isolated from clinical samples (Ramírez et al., 2010; Di Noto et al., 2016; Almuzara et al., 2017). Few studies are limited and focused on the analysis of the accessory genome of *Shewanella* spp., particularly on the mobilome, resistome, and virulome of *S. algae* isolates (Deng et al., 2019; Wang et al., 2020; Huang et al., 2022), which encode beneficial genetic traits that contribute to its survival. Recently, Zhong et al. (2018) suggested that *Shewanella* spp. can acquire different mechanisms by HGT, in order to adapt to diverse environments.

In addition, the mobilome of *S. algae* and *Shewanella baltica* were further analyzed revealing the presence of strain-specific mobile genetic elements (MGEs) (Uhrynowski et al., 2019; Zago et al., 2020). Although these reports provided helpful information on the accessory genome of this genus, no studies have addressed an integrated analysis. Here, we performed an in-depth comparative study of the accessory genome focusing on the mobilome, resistome, and virulome. We found that infections caused by *Shewanella* spp. are not restricted to these two species, but virulence factors are distributed in two main lineages. Taken together, our results suggest that the genus *Shewanella* has the capability to evolve as a threat if correct identification and surveillance is not taken into consideration.

## 2. Materials and methods

### 2.1. Data retrieval and panmatrix analysis

**FIGURE 1:**
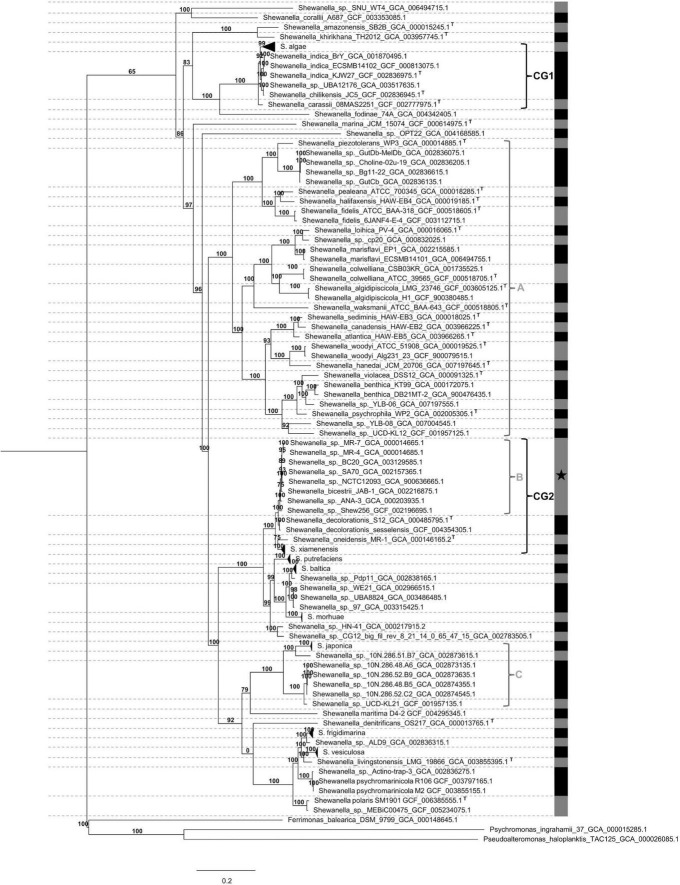
Collapsed phylogenetic tree of *Shewanella* spp. Pairwise average nucleotide identity (ANI) was estimated for all genomes. Dotted horizontal lines (- - -) separate monophyletic groups; numbers above branches indicate bootstraps support; vertical gray and black lines show ANI values; letter T denotes type-strain isolates; black star (*) indicates species with marginal ANI values; horizontal triangles in branches depict collapsed lineages; letters A, B, and C indicate monophyletic and polyphyletic lineages; CG1 (clinical group 1) and CG2 (clinical group 2) represent monophyletic groups with 100% statistical node support containing clinical isolates; NCG (non-clinical group) encompasses remaining genomes. *Alteromonadales* genomes were used as outgroups.

Complete and draft genomes of *Shewanella* spp. available in Genbank until August 2019 were used in this work, resulting in 144 sequences (GCA and GCF accession numbers; Supplementary Table 1). Type-strains were included in this study and identified with a T in Figure 1 and Supplementary Figure 2. Orthologous genes were identified by the Get_homologues software (Contreras-Moreira and Vinuesa, 2013) using the OrthoMCL method. A minimum coverage of 75% and identity values of 40% were set as thresholds for blastp searches. GFF, FAA, and FNA files of all genomes used in this work are available at https://figshare.com/authors/QuirogaLab_IMPaM_UBA-CONICET_/14600396.

### 2.2. Phylogenetic analysis

Amino acid sequences from orthologous genes were aligned using Clustal Omega v1.2.0 and concatenated using local developed scripts available at (https://github.com/LBC-Iriarte/Shewanella_genomics). The final dataset comprised 20,931 aligned positions. Model selection and phylogenetic analyses were done using IQ-Tree v1.6.12 (Nguyen et al., 2015), and support for the nodes was evaluated with the ultrafast bootstrap procedure.

### 2.3. Average nucleotide identity

Average nucleotide identity (ANI) with values equal or higher than 95% were assigned to the same species (Konstantinidis and Tiedje, 2005; Goris et al., 2007). The two-way ANI, reciprocal best hits-based comparison, was estimated using the ani.rb script (Supplementary Table 2).

### 2.4. Accessory genome analysis

We used the identification of the *rep* gene for plasmid detection as described previously (Cameranesi et al., 2017). The similarity search was done using tblastn, blastp, and psi-blast, selecting reference replicase genes of plasmids reported in *Shewanella* spp. genomes as query (Supplementary Table 3). Rep sequences were employed for multiple sequence alignment with ClustalW in MEGA X (Kumar et al., 2018) and tree construction as described above. Replicases from other genera and incompatibility groups were included in the analysis. In addition, blastn searches with complete plasmid sequences were also used as queries against our dataset.

Insertion sequences (ISs) were identified using *VR*profile 2.0 (Li et al., 2018) and ISFinder (Siguier, 2006). Putative ISs were classified as: (i) “complete ISs” to those that have a full-length sequence, a score >1,000, identity >90%, and an *H*-value >0.9, (ii) “IS-like elements” to those with a score <1,000, identity between 90 and 80%, and *H*-value between 0.9 and 0.8, and (iii) “IS pseudogenes” to those with a score <1,000, identity <80%, and *H*-value <0.8. Only complete ISs and IS-like elements were included in our analyses (Li et al., 2018).

Mobile integrons were detected using blastp with integrase sequences IntI1 (ADW78905.1), IntI2 (ADH82153.1), IntI3 (AAO32355.1), IntI4 (AAD53319.1), and IntI5 (AAD55407.2) as queries. Candidates with an identity value above 95% were further considered. Classification of integrons was done based on the INTEGRALL database (Moura et al., 2009).

Group II introns were detected by blastp using a reference maturase sequence (AAL51020.1) as a query following previous published proceedings (Quiroga and Centrón, 2009). Candidate maturases having an identity value >45% and the characteristic YADD motif in their sequences were included in the study.

Genomic islands (GIs) were detected using Islandviewer 4 (Bertelli et al., 2017) and AlienHunter V.1.7-10 (Vernikos and Parkhill, 2006) softwares.

Identification of prophages was done using the program PHASTER (Arndt et al., 2016). Putative phages with score >70 were included in the analysis, and classified as intact or questionable elements.

Integrative and conjugative elements from the SXT/R391 family were identified by means of blastn, using the gene *traV* (accession number LGYY01000082.1) from *S. xiamenensis* Sh95 as query. Sequences with nucleotide identity >95% and coverage >95% were included in the analysis. The presence of full-length ICE structure was also verified by blastn. Artemis Comparison Tool (ACT) (Carver et al., 2005) was used for the analysis of all GIs and the identification of the respective genetic locus.

CRISPRCasFinder (Couvin et al., 2018) was used to detect CRISPR (clustered regularly interspaced short palindromic repeats) arrays and Cas proteins. Curation of CRISPR-Cas types was done following a previously implemented criteria (Makarova et al., 2020).

Antimicrobial resistance genes were detected using ResFinder 4.1 (Bortolaia et al., 2020) and the RGI (Resistance Gene Identifier) (Alcock et al., 2020) tool from CARD (The Comprehensive Antibiotic Resistance Database) with their respective default parameters.

Virulome was obtained by searching for virulence factor genes using the tool VFanalyzer from VFDB (Liu et al., 2022) with default parameters. Since there is no database for the genus *Shewanella*, we compared genomes against *Pseudomonas* and *Vibrio* spp. virulence factor databases, which can cause infection in humans and also thrive in aquatic niches.

### 2.5. GO term enrichment analysis

Functional annotation of *Shewanella* spp. genomes was carried out by eggNOG-mapper v2 using the Diamond search mode (Cantalapiedra et al., 2021). An *e*-value <0.001, protein sequence identity >30%, and a query coverage >70% were set as the threshold for homology searches. To perform the Gene-Set Enrichment Analysis (GSEA), we constructed a presence-absence matrix of all the Gene Ontology (GO) terms present in the genus. Then, the Fisher’s exact test was applied to identify significantly enriched functional terms (*p*–value <0.05) in the “clinical” groups (CGs) respective to the “non-clinical” group (NCG) (Supplementary Table 4). CGs were independently compared to each other and against the NCG (Supplementary Tables 5, 6). The Fisher test was performed using the “stats” package from the R core team. The GO term annotations of genes were handled using GOATOOLS (Klopfenstein et al., 2018; Supplementary Tables 7, 8). For each enrichment test the phi-coefficient of correlation was calculated. In order to support the functional annotation, blastp searches against the original genome database were done using genes annotated within significantly overrepresented functional terms as queries (Supplementary Table 8). The phylogenetic distribution study and sequence analysis of homologous genes in CGs and NCG were done. A blastp search was performed against PROSITE (Sigrist et al., 2012) and PFam v. 35.0 databases (Mistry et al., 2021) to assess the presence of conserved protein motifs. The workflow of this section is shown in Supplementary Figure 1. Script is available at https://github.com/LBC-Iriarte/Shewanella_genomics.

## 3. Results

### 3.1. Characterization of *Shewanella* lineages

Our dataset comprised 144 genomes from *Shewanella* spp. isolates recovered from different niches. Their evolutionary relationship was established based on a maximum likelihood phylogenetic tree analysis of 78 concatenated orthologous genes. Using ANI values (Supplementary Table 2) and known type-strains as reference, we identified several species distributed in polyphyletic and monophyletic groups, which were consistent with a previous report that recommended the revision of *Shewanella*’s taxonomy (Figure 1 and Supplementary Figure 2; Thorell et al., 2019). Species assignment revealed marginally significant ANI values (94% < ANI < 96%) for the cluster encompassing strains MR-7, MR-4, BC20, SA70, NCTC12093, JAB-1, ANA-3, and Shew256 (Supplementary Table 2, Figures 1, and Supplementary Figure 2, Cluster B, marked with a star [*]). This result suggests that a speciation process is most likely in progress, thus, the definition of species in this group based on whole genome sequence comparison may improve once including additional genomes. Revision of the species classification revealed that three strains previously identified as *S. putrefaciens* (SA70, NCTC12093, and 97) were not grouped in the corresponding lineage, thus we suggest renaming them as indicated in Table 1. Furthermore, we propose a re-assignation of strains *Shewanella*. sp. FDAARGOS 354, LC6, LC2, *Shewanella oneidensis* S2 and POL2, to *S. xiamenensis*; and, strains CG_18_big_fil_WC_8_21_14_2_50_42_11, CG_4_9_14 _0_8_um_filter_42_14, CG_4_10_14_0_8_um_filter_42_13, and CG_4_10_14_3_umfilter_42_91 to *Shewanella vesiculosa* (Table 1).

**TABLE 1:** Re-assignment of *Shewanella* spp. strains.

Linage	Strain	Species re-assignation
Cluster B	*Shewanella putrefaciens* SA70	*Shewanella* sp. SA70
	*Shewanella putrefaciens* NCTC12093	*Shewanella* sp. NCTC12093
*S. xiamenensis*	*Shewanella* sp. FDAARGOS 354	*Shewanella xiamenensis* FDAARGOS 354
	*Shewanella* sp. LC6	*Shewanella xiamenensis* LC6
	*Shewanella* sp. LC2	*Shewanella xiamenensis* LC2
	*Shewanella oneidensis* S2_009_000_R2_72	*Shewanella xiamenensis* S2_009_000_R2_72
	*Shewanella* sp. ZOR0012	*Shewanella xiamenensis* ZOR0012
	*Shewanella* sp. POL2	*Shewanella xiamenensis* POL2
*Shewanella putrefaciens* 97	*Shewanella* sp. 97
*S. vesiculosa*	*Shewanella* sp. CG18 big fil WC 8 21 14 2 50 42 11	*Shewanella vesiculosa* CG18 big fil WC 8 21 14 2 50 42 11
	*Shewanella* sp. CG 4 9 14 0 8 um filter 42 14	*Shewanella vesiculosa* CG 4 9 14 0 8 um filter 42 14
	*Shewanella* sp. CG 4 10 14 0 8 um filter 42 13	*Shewanella vesiculosa* CG 4 10 14 0 8 um filter 42 13
	*Shewanella* sp. CG 4 10 14 3 um filter 42 91	*Shewanella vesiculosa* CG 4 10 14 3 um filter 42 91

Our analysis showed that infections caused by *Shewanella* species are not restricted to *S. algae* (Supplementary Figure 2). Accordingly, the group comprising Cluster B and lineages *Shewanella decolorationis* and *S. xiamenensis* contain isolates recovered from clinical samples responsible for causing skin and soft-tissue infections, biliary tract infections, peritonitis, and ocular infections (Supplementary Figure 2). Noteworthy, after revision and reassignment of species from our dataset based on the phylogenomics and ANI analyses, we observed that *S. putrefaciens* isolates were recovered from either environmental niches, animal-host, oil production or effluents, and none of them were responsible for human-associated infections. This suggests that *S. putrefaciens* strains have been misidentified and overestimated as an opportunistic pathogen. Further studies are necessary to confirm whether *S. putrefaciens* is truly responsible for causing infections in humans.

### 3.2. Mobilome analysis of *Shewanella* genomes

#### 3.2.1. Circulating plasmids among *Shewanella* spp.

**FIGURE 2:**
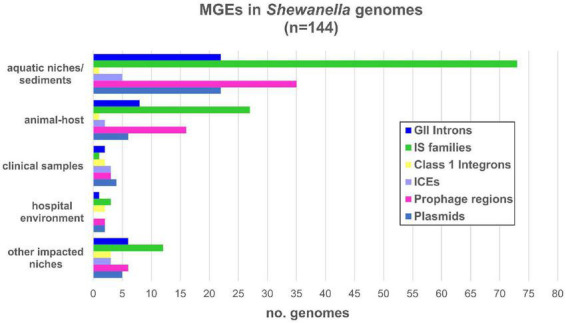
Mobile genetic elements detected in *Shewanella* spp. from different niches. Number of MGE types detected in each genome: GII introns (group II introns); IS families (insertion sequence families) listed in Supplementary Table 10; IntI: mobile class 1 integrons; ICE (integrative and conjugative elements); prophage regions with intact and questionable scores listed in Supplementary Table 11; plasmids listed in Supplementary Table 9.

*Shewanella* harbors a wide variety of MGEs such as plasmids, IS, transposons, phages, ICE, among others (Figure 2). In order to identify plasmids in the analyzed genomes, we looked for previously identified Rep sequences (Supplementary Table 2). We observed that several species contained one or more plasmids in their genome (42/144; Supplementary Figure 2 and Supplementary Table 9). The estimated plasmid size ranged from a few kb (∼4.9 kb; NZ_CM009108.1) to up to ∼355 kp (NZ_CP043903.1), with 10 plasmids with a size above 100 kb.

Eleven genomes contained more than two plasmids (Supplementary Table 9). Cluster B and lineages *S. decolorationis* and *S. xiamenensis* showed the higher occurrence with up to two plasmids in each genome. Although previous studies reported up to four plasmids in *S. baltica* lineage (Brettar et al., 2001; Vogel et al., 2005; Caro-Quintero et al., 2012), we identified their respective replicases in only a few of them.

**FIGURE 3:**
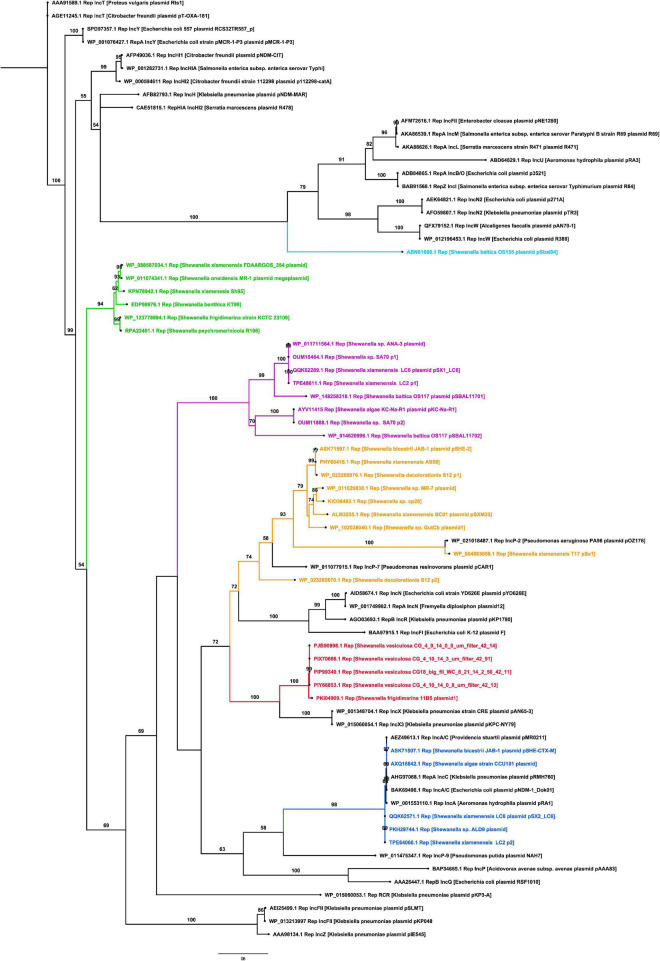
Phylogenetic tree of replicases found in *Shewanella* spp. genomes. The maximum likelihood tree was constructed using IQ-TREE v1.6.12 with 1,000 bootstraps. Numbers above branches indicate bootstrap support (%). Branches in red contain plasmids related to IncX incompatibility group, branches in blue contain plasmids related to the IncA/C group, branches in yellow contain plasmids related to IncP group, branches in green and purple comprise independent clusters of unknown replicases, and black branches depict known replicases.

The replicase phylogenetic tree showed that identified plasmids belong to different incompatibility groups (Figure 3). Several replicases found in *Shewanella* plasmids were closely related to IncA/C, IncX, and IncP (Figure 3), commonly found in *Pseudomonas* spp. and *Enterobacteriaceae* isolated from clinical samples (Carattoli, 2013). Plasmids like pSx1 (IncP) and pSHE-CTX-M (IncA/C) may be capable of replicating in those bacteria. These incompatibility groups are known for harboring antimicrobial resistance genes (ARG), reflecting their participation in the AMR evolution and the potential ability of *Shewanella* to acquire new determinants.

The remaining replicases, which clustered together in two groups (Figure 3), are encoded in plasmids spread among different *Shewanella* lineages, i.e., plasmids pKC-Na-R1 from *S. algae*, pSBAL11701 from *S. baltica*, and pSX1_LC6 from *S. xiamenensis*. This phylogenetic patchy distribution was also observed in *Shewanella* plasmids related to IncA/C and IncX groups (Figure 3).

When we analyzed the incidence of plasmids based on their source, we observed that almost half of the human-related isolates (from clinical samples, other impacted areas, or hospital environments) contained at least one of these elements (10/23; 43.47%) (Figure 2 and Supplementary Tables 1, 9). Conversely, fewer plasmids were found in genomes from animal host-bacteria (8/33; 24.24%) and from aquatic or sediment niches (23/82; 28.05%).

Taken together, our results showed that there is a high versatility and variability of plasmids circulating in *Shewanella* species, which are widely spread among the different phylogenetic groups. Their close relationship with plasmids circulating in clinical isolates suggest a probable genetic exchange that may contribute to the evolution of *Shewanella* genomes and the acquisition of selected traits.

#### 3.2.2. Insertion sequences encoded in *Shewanella* spp. genomes

**FIGURE 4:**
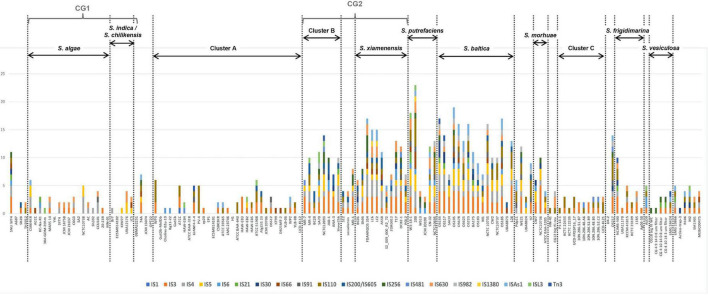
Insertion sequence families found in *Shewanella* spp. genomes. Genomes (*n* = 144) were sorted according to the phylogenetic tree. Vertical dotted lines separate the different lineages and clusters. *Y*-axis depicts the number of non-redundant ISs (*n* = 178) found in *Shewanella* spp. genomes. Colored bars represent each IS family (*n* = 19); each family can have >1 IS (Supplementary Table 10).

We searched for known ISs in *Shewanella* spp. genomes using freely available data. We detected 19 IS families and 178 different ISs (Supplementary Table 10). The most frequent ISs found in *Shewanella* genomes belonged to families IS*3* (IS*Sba4*: *n* = 24; IS*Sba5*: *n* = 20), IS*4* (IS*Sba6*: *n* = 25), IS*110* (IS*Sod19*: *n* = 23), and IS*982* (IS*Sod20*: *n* = 21) (Supplementary Figure 3A and Supplementary Table 10). The analysis of the occurrence of IS families (ISfam) among lineages showed a higher incidence in *S. xiamenensis* (*n* = 5–9 ISfam/genome), *S. putrefaciens* (*n* = 4–12 ISfam/genome), and *S. baltica* (*n* = 4–12 ISfam/genome; except for strain UBA8873 with only 2 IS families), and in Cluster B (*n* = 3–9 ISfam/genome) (Figure 4). Since these species are closely related, it is possible to assume that similar cellular factors contribute to IS activity and dissemination in their genomes.

We found that four genomes contained more than 100 ISs (including identical copies), and most of them belonged to the *S. baltica* lineage (Supplementary Table 10). *S. baltica* OS117, *S. oneidensis* MR-1, and *S. baltica* OS155 had the highest number of ISs with 124, 121, and 120 elements, respectively (Supplementary Table 10). Sixteen genomes did not show complete IS or IS-like elements; since 14 of them were draft sequences, this may explain their absence. Moreover, we did not observe a difference in the number of ISfam/genome among the different niches (Supplementary Figure 3B). This suggests that there may not be an association between the source of an isolate and the spread of these elements (Supplementary Table 10). In addition, we did not find an association between plasmid presence and IS incidence.

#### 3.2.3. Mobile integrons and group II introns found in *Shewanella* spp. genomes

A recent work showed the diversity of integron integrases in the genus (Nuñez et al., 2022). We focused the analysis on our dataset where we found the *intI1* integrase gene in eight genomes (Supplementary Figure 2). Analysis of the genetic content of those class 1 integrons revealed different cassette arrays and MGE associations. Strain *S. baltica* CW2 had a complex class 1 integron in the chromosome. Strain KC-Na-R1 contained two integrons located in plasmid pKC-Na-R1: In469 (Zhu et al., 2020) and a class 1 integron which harbors a similar variable region reported in In622 (Supplementary Figure 4). The structure of the integron In622 was consistent with a complex class 1 integron, since it had a second variable region with the *qnrA* gene. Strain *S. xiamenensis* T17 harbored three class 1 integrons embedded in Tn*6297* transposon in plasmid pSx1, which corresponded to In27-like, In1357, and a complex class 1 integron (In4-like) (Yousfi et al., 2017). Three other genomes contained a single integron carrying *aadA2* gene cassette (strains LC2, LC6, and ALD9), whereas strain Shew256 had a gene cassette array consisting of *arr3-dfrA27-aadA16* (Almuzara et al., 2017; Supplementary Figure 4). Furthermore, strain LC6 contained a class 1 integron within plasmid pSX1_LC6, which shared 99.98% with a contig that had the class 1 integron from strain LC2 (VFSJ01000026.1), suggesting that both isolates harbored a similar plasmid. Regarding the *intI9*-like gene, aside from the previously reported integron in *S. xiamenensis* Sh95 (Parmeciano Di Noto et al., 2016), no other genome encoded this genetic element.

With regards to the source of bacteria that host these class 1 integrons, we did not find any correlation. These integrons were found either in clinical samples (*n* = 1), hospital environments (*n* = 2), effluents (*n* = 3), aquatic animal hosts (*n* = 1), or aquatic niches (*n* = 1) (Figure 2). The limited distribution of integrons and their location in MGEs reflects their acquisition by HGT events; however, further analyses are necessary to identify the platforms involved in their dissemination.

Previous studies have shown the incidence of group II (GII) introns in *Shewanella* genomes (Quiroga and Centrón, 2009). Thus, we looked for this MGE using the typical maturase sequence with blastp. As result, we found that 42/144 (29.16%) genomes contained a GII intron (Supplementary Figure 2). These retroelements were distributed in all lineages, with a higher incidence in some of them, e.g., *S. xiamenensis* and *S. baltica* (Supplementary Figure 2). The occurrence of GII introns based on the niche shows that these elements were found in 31 samples from environmental or animal-associated niches (73.81%), 10 in human-related niches (23.81%), and 3 in bacteria of unknown origin. Noteworthy, a subclass of GII introns, identified as class C-attC, can invade the gene cassettes of integrons at the *attC* sites (Quiroga et al., 2008), which may contribute in their occurrence among human-associated *Shewanella* spp. isolates. Accordingly, class 1 integrons from strains CW2 and KC-Na-R1 contained these ribozymes inserted within their respective variable regions (Supplementary Figure 4).

#### 3.2.4. Prophages, SXT/R391 ICEs, and other genomic islands present in *Shewanella* spp.

Detection of prophages showed that almost all genomes contained, either the complete element (*n* = 36), partial regions (*n* = 20), or phage-related proteins (*n* = 80) (Supplementary Table 11). While most genomes contained one or two copies of intact or partial prophages, we found all six intact prophages in *S. oneidensis* MR-1 previously reported (Rodionov et al., 2011). Complete and partial prophages were found in all lineages (Supplementary Figure 2), which indicates that there is no correlation between the distribution of these elements and specific *Shewanella* spp. Taking into account the source of each isolate, we assessed their incidence, ranging from 40 to 47%, which indicates a similar occurrence of prophages in *Shewanella* strains from different habitats.

On the other hand, we searched for ICEs from the SXT/R391 family since they have been previously reported in this genus (Pembroke and Piterina, 2006; Parmeciano Di Noto et al., 2016, 2019; Fang et al., 2018). We found that only 15 genomes contained these elements. These ICEs were found in clinical and environmental samples alike, showing no direct bias toward their source nor with any specific lineage (Figure 2 and Supplementary Figure 2). These platforms carried all necessary core genes (i.e., *tra* and *set* operons, and *int/xis* and *bet/exo* modules) for their activity and dissemination to other bacterial hosts (Parmeciano Di Noto et al., 2016). A significant diversity among the variable regions or hotspots of these ICE were found, except for strains *S. xiamenensis* LC6 and LC2, and *S. vesiculosa* CG18, CG4_9_14_0_8, CG4_10_14_0_8, and CG4_10_14_3. We noted that the last three strains form a monophyletic group with ANI values >99.95% (Supplementary Figure 2). The insertion of ICE SXT/R391 in *Shewanella* spp. occurred at the *prfC* gene, except for strain Sh95 which was found at the *pabA locus* as a result of encoding a unique *int/xis* module (Parmeciano Di Noto et al., 2016).

Last, we did an exploratory analysis of the remaining identified GIs focusing solely on complete genomes to avoid a misidentification due to partial sequences. *Shewanella* spp. genomes contained several islands with sizes larger than 10 kb and spanning up to 100 kb. On average, members of this genus have 14 GIs (ranging from 3 to 27; Supplementary Figure 2) comprising repair/recombination/partitioning genes (*xerC*, *par*, and *radC*), virulence genes (*pil, flp*, *fli, flg*, and *hly)*, and TA systems (*relE/parE*, *hicA/hicB*, and *doc/phD*), suggesting that they may correspond to pathogenicity islands with a potential transferability. Further analyses are necessary to define their function.

### 3.3. CRISPR-Cas systems in *Shewanella* spp. genomes

**FIGURE 5:**
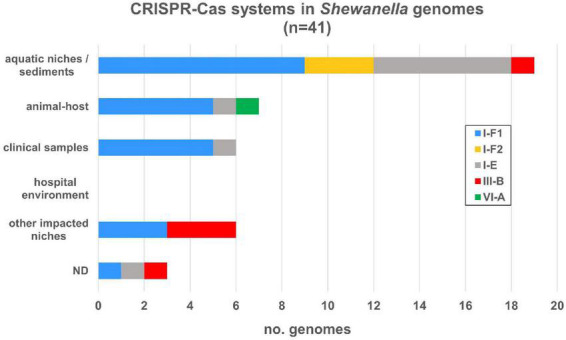
CRISPR-Cas systems identified in *Shewanella* spp. genomes from different niches. CRISPR-Cas systems were depicted according to the source of each strain. Colored bars represent each subtype (I-E, I-F1, I-F2, III-B, and VI-A) (Supplementary Table 12).

Among the islands detected in our research we found several CRISPR-Cas systems, thus we expanded our analysis and search for these defense elements. We evaluated their occurrence in this genus and we were able to identify four types of CRISPR-Cas systems: I-E, I-F, III-B, and VI-A, in 41 genomes, each with their respective *cas* operon (Supplementary Figure 2 and Supplementary Table 12). The most frequent system (27/41; 65.85%) corresponded to the I-F type and, although its distribution was scattered in all lineages, most of them were found in *S. algae* and *S. putrefaciens*. Among I-F systems we found two variants: the variant I-F1 that carries all *cas* genes, and the smaller variant I-F2, which lacks *cas8f* gene (Makarova et al., 2020). Type I-E system was found in nine genomes (21.95%) in different lineages, whereas type III-B was only found in three *S. xiamenensis* strains, in *S. putrefaciens* 200 and in *S. baltica* OS625. A single type VI-A system was detected in this dataset (*Shewanella japonica* P1-14-1). While all genomes in our dataset contained one operon *cas* system with its respective arrays, *S. putrefaciens* 200 had two CRISPR-Cas systems: III-B and I-F2 types (Supplementary Table 12). On the other hand, we also observed a heterogeneous distribution of I-F, I-E, and III-B systems in all niches (Figure 5).

Regarding the CRISPR arrays associated with each *cas* operon, we found that 34 (82.93%) systems contained a single array and most of them have a significant number of CRISPRs. Type I-F arrays contained up to 153 CRISPRs, type I-E from 1 to 143, and type III-B up to 37. No arrays were found associated with the type VI-A system. On average, type I-F systems had 51 CRISPRs with a median of 43. *S. xiamenensis* Sh95 (*n* = 153) contained the maximum number of CRISPRs in a type I-F1 system (Supplementary Figure 2). Type I-E systems had on average 40.55 CRISPRs (median of 40.5) and the genome with the largest number of arrays corresponded to *Shewanella* sp. CG12 big_fil_rev_8_21_14_0_65_47_15 (*n* = 143). Type III-B had on average 31 CRISPR arrays. It is possible to assume that CRISPR-Cas systems carrying arrays with >40 CRISPRs are active defense systems capable of adaptation upon further invasions. We also found arrays distant from the *cas* operon that can also contribute to the host defense machinery (data not shown). Overall, the incidence of complete CRISPR-Cas systems in this genus is 28.47%, where almost all bacteria harboring these defense mechanisms carry the *cas* genes with at least one array in the vicinity. In addition, we did not observe a definitive association with a specific niche nor species.

### 3.4. Resistome analysis of *Shewanella* spp.

**FIGURE 6:**
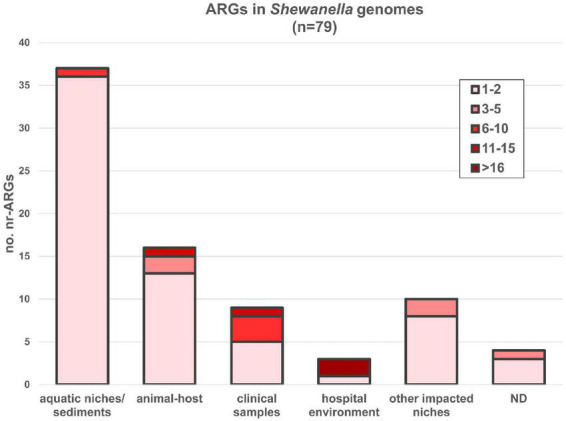
ARGs found in *Shewanella* spp. genomes from different niches. AMR genes were depicted according to the source of each strain. nr-ARG represents non-redundant ARM genes in genomes. Colored gradient depicts the range of genes encoded in each genome (Supplementary Table 13).

Seventy-nine out of 144 genomes (54.86%) encoded at least one ARG susceptible to HGT (Figure 6 and Supplementary Table 13). Most bacteria carrying these genes were isolated from aquatic niches or sediments (*n* = 37); however, bacteria carrying resistance genes were also recovered from clinical samples (*n* = 8), human-related niches (*n* = 13; hospital and other environments), or different animal hosts (*n* = 16). Bacteria isolated from hospital environments showed the highest accumulation of ARGs, reaching up to 19 resistance determinants in *S. xiamenensis* T17 which was recovered from a nosocomial effluent in Algeria (Supplementary Tables 1, 13). Furthermore, 11 genomes encoded 3 or more different resistance mechanisms resulting in multidrug or extensively drug resistant bacteria (Supplementary Table 13, marked with an asterisk). Among them, eight bacteria were isolated from human-related sources, whereas two were recovered from marine animals and one from the sea ice near Alaska.

Incidence of ARGs was highest in *S. algae, S. xiamenensis*, and in Cluster B, where all members encoded at least one resistance mechanism (Supplementary Figure 2 and Supplementary Table 13). In this regard, it has been previously reported the association of *bla*_OXA–48_ and *bla*_OXA–55_ variants with *S. xiamenensis* and *S. algae*, respectively (Tacão et al., 2018; Ohama et al., 2021). Our global analysis was consistent with this report. All isolates from the monophyletic group spanning from Cluster B to *S. xiamenensis* lineage possessed variants of the carbapenem resistant gene *bla*_OXA–48_, whereas *bla*_OXA–55_-like genes were only found in *S. algae*. However, recent revision of the OXA classification (Naas et al., 2017) showed that there were 11 OXA-48 variants, whereas the OXA-55 variants corresponded to OXA-729. In addition, the recently reported beta-lactamase gene *bla*_OXA–900_ (Frenk et al., 2021) was detected in all genomes from the *S. putrefaciens* lineage showing a probable species-specific association similar to those reported for variants OXA-48-like and OXA-55-like.

Our analysis also confirmed the association between the lineage *S. algae* and the quinolone resistance determinant *qnrA* (Araújo et al., 2021). All *S. algae* genomes contained a variant confirming this lineage acts as a gene reservoir. Moreover, three other isolates, *Shewanella* sp. SA70, *Shewanella* sp. Shew256, and *S. xiamenensis* T17, encoded other *qnr* determinants. Strain SA70 contained a *qnrA1* variant in a complex class 1 integron (In1080) and a *qnrVC1* gene in its chromosome (Supplementary Table 13 and Supplementary Figure 4). On the other hand, variant *qnrVC6* was detected in the clinical sample *Shewanella* sp. Shew256, and in *S. xiamenensis* T17 that was recovered from hospital settings. On the contrary, *qnrVC* variants were found embedded in MGEs in human related-MDR bacteria, which indicates that HGT may have been involved in their dissemination. We thus suggest that while some specific *Shewanella* spp. are reservoirs of resistance determinants, others may adapt and acquire homologous genes in order to survive in the environment.

Furthermore, plasmid-mediated colistin-resistant genes (*mcr*) were reported previously in this genus (Zhang et al., 2019a). Only five of them encoded the colistin resistant determinant *mcr-4.3*: 3 strains from the *S. vesiculosa* lineage, 1 strain from the *Shewanella frigidimarina* lineage and in the isolate *Shewanella* sp. SNU WT4. We did not find an association among these genes and a specific lineage or plasmid. Since these genes were not conserved in any lineage, further studies are necessary to determine the role of this bacterium as a possible *mcr* reservoir.

Other ARGs that confer resistance to aminoglycosides (*aac* and *aph*), sulfonamides (*sul2*), tetracyclines (*tetA, tetD*, and *tetG*), chloramphenicol (*floR*, *cmx*, and *catB11*), and other beta-lactams (*bla*_SLB–1_, *bla*_SFB–1_, *bla*_CARB3_, and *bla*_CTX–M–15_), were found in a few genomes (Supplementary Table 13). Noteworthy, the clinical isolate *Shewanella bicestrii* JAB-1 encoded 10 ARGs, most of them harbored in the plasmid pSHE-CTX-M where they were adjacent to or nearby different ISs (Jousset et al., 2018).

### 3.5. Virulome analysis of *Shewanella* spp.

To date, the virulome of the genus *Shewanella* has not been fully characterized. In order to identify their virulence genes, we recovered the data using *Pseudomonas* and *Vibrio* databases, which can thrive in aquatic niches and cause infection in humans. We obtained 360 putative genes encoding for fimbriae, hemolysins, flagella, secretion systems, autoinducers, toxins, adhesins, siderophores, capsule, among others (Supplementary Table 14 and Supplementary Figure 5), with an average of 106 candidate genes per genome. Most bacteria contained genes for chemotaxis/motility, LPS and capsule synthesis, type IV pilus, biofilm formation, quorum sensing, pyoverdine receptors, iron transporter, heme biosynthesis, EPS T2SS, phytotoxin, and *hlyA* toxin.

Whereas we did not observe an association between *Shewanella* spp. clinical isolates and a specific group of virulence factors analyzed, we noticed a correlation between the VAS T6SS and the *S. algae* lineage (Supplementary Figure 5). This system was present in almost all *S. algae* genomes and a few isolates from Cluster A, which were recovered from different sources (Supplementary Table 14). In addition, T3SS genes were detected in seven genomes from strains isolated either from aquatic niches, sediments, or marine animals, evidencing a lack of correlation of the secretion systems with the host source.

Furthermore, several genes showed a higher incidence in specific lineages, such as *irgA* (iron-regulated adhesin), *lasB* (elastase), and *zmp1* (Zn-metalloprotease) homologs, which were detected mostly in *S. algae* and *S. xiamenensis*. We also observed that a few isolates from a variety of sources encoded the invasin IbeB, which participates in cell invasion. Taken together our analysis suggests that all *Shewanella* species encode different virulence traits that may help them to thrive and adapt to different environments and hosts. Notwithstanding, the T6SSs may be a key virulence system that contributes to *S. algae* virulence.

### 3.6. Functional enrichment test and paralogs genes associated with clinical lineages

#### 3.6.1. Functional terms significantly over and under-represented within clinical groups

Strategies based on the detection of conserved virulence genes led to the identification of a few candidates that may explain the pathogenicity of *Shewanella* spp. Although this approach provided useful information, we also used an alternative strategy based on GSEA, which resulted in the recognition of functional GO terms and its associated genes that may be involved in their virulence (Supplementary Figure 1). We defined two lineages that contained all isolates that cause infection in humans: “clinical group 1” (CG1) and “clinical group 2” (CG2) (Supplementary Figure 2). Not all the isolates within those two groups were clinical nor human-related samples but both CGs were the more inclusive and statistically supported monophyletic groups containing clinical isolates (Figure 1 and Supplementary Figure 2). A total of 374 functional GO terms were found enriched in the CGs vs. NCG (non-clinical group, which encompasses the remaining lineages) (Supplementary Table 4). Independent comparison of each CG against NCG resulted in 331 and 273 terms enriched in the CG1 and CG2, respectively (Supplementary Tables 5, 6). CG1 and CG2 shared 189 GO terms, which could indicate a convergent evolution. Several GO terms were associated with virulence, which included genes associated with motility (*ycdX*, *yeaJ*, and *cheA*), iron metabolism (*ftnA*, *hmuR*, and *tuf*), processes associated with oxygen free radicals (*katG*), cobalamin biosynthesis (*cobS*), T6SS (*vgrG1*), T2SS (*gpsK*), and polyamines (*speCEG* and *potE*) (Supplementary Tables 7, 8). Some functional GO terms associated with AMR were also enriched in CGs. Linked to those terms we identify a few genes, such as, *ampC* (beta-lactams), *emrE* (aminoglycosides and aromatic compounds efflux pump), *folM* (trimethoprim-sulfamethoxazole), *folX* (trimethoprim-sulfamethoxazole), *nagA* (chloramphenicol), *nfnB* (nitrofuran and nitrofurantoin), and *rfbC* (antimicrobial peptides). These results show that this approach complements previous data and contributes to the search for virulence and ARGs.

#### 3.6.2. Paralogous genes associated with the clinical niche

The GSEA analysis led us to identify the presence of two copies of *katG* in 44 genomes: 14 in CG1, 20 in CG2, and 10 in NCGs. Sequence analysis of both genes (*katG_1* and *katG_2)* showed an average of 56% identity and 95% coverage at the amino acid level, with the highest conservation within the CGs. Since both genes had the same protein family profiles, we were able to infer that they carried on similar functions (Supplementary Figure 6A, in blue). The analysis of their distribution showed that *katG_1* was encoded in almost all CG1 and CG2 genomes and in a few NCG genomes, whereas *katG_2* showed a wider distribution in the genus (Supplementary Table 8 and Supplementary Figure 6B).

Manual inspection of enriched GO terms led us to identify four other highly diverged paralogs genes (amino acid identity ranging from 30 to 45%) that shared the same conserved functional domain. These genes corresponded to *cysQ*, *hemB*, *kefB*, and *mod*A (Supplementary Figure 7). The phylogenetic distribution of these genes in CG1, CG2, and NCG showed that in all cases, one homologous gene of *hemB*, *kefB*, and *modA* was significantly more frequent in the CGs, while the other copy was widely distributed in the genus. On the other hand, the *cysQ* gene belonged to a functional GO term enriched in CG genomes, but the frequency of both paralogs was also high in NCG genomes (Supplementary Tables 7, 8).

## 4. Discussion

*Shewanella* genus has a versatile and diverse accessory genome that contributes to its adaptation and survival to different niches. This genus showed a wide variety of genes related to the mobilome, the virulome, and the resistome scattered heterogeneously throughout all lineages and habitats. In-depth analysis of these genetic traits in a large set of genomes from a wide variety of species proceeding from different niches revealed a few associations that may provide new insights into the pathogenicity and evolution of *Shewanella*.

*Shewanella* mobilome consists of various MGEs widely distributed along all lineages. The comparative analysis of lineages with more representation in each cluster, such as *S. algae*, *S. xiamenensis*, and *S. baltica*, reflected the MGE diversity within species and the plastic nature of the *Shewanella* genome. A previous work suggested that the rate at which HGT happens is up to two orders of magnitude higher than per-gene point mutations (Puigbò et al., 2014), which indicates that the gene content of microbes can vary in relatively short timescales. MGEs found in this genus were quite diverse, reflecting their continuous evolution, contributing with the adaptation and survival of the host to different environments. Among the MGEs detected, ISs showed the broadest diversity with the highest variety and copy number per element within the CG2 group, *S. putrefaciens*, and *S. baltica* (Supplementary Figure 2). It is well known that ISs can collaborate with the reshaping and rearranging of bacterial genomes, but it is less clear which factors govern their dissemination throughout a genome and how it impacts on the host physiology and evolution (Vandecraen et al., 2017). It is likely that the target site availability is a major contributor; however, interference with other ISs or host factors may be limiting features specific to each lineage (Siguier et al., 2014). A similar pattern was observed for GII introns, which have been previously found in some *Shewanella* spp. genomes (Quiroga and Centrón, 2009) although their distribution was not related to the source or the lineage. Incidence of ISs and GII introns was lower in CG1 and Cluster A, suggesting that a common ancestor to lineages encompassing species from Cluster B up to Cluster C may have acquired some of these MGEs (such as, IS*Sod19*, IS*Spu8*, IS*Sod20*, IS*Sba4*, and IS*Sba5*) and maintained them thereafter (Supplementary Table 10).

On the other hand, prophages were heterogeneously distributed in all lineages regardless of their source, where a single genome may contain up to six different prophages. We did not observe their conservation within specific lineages, which may be explained by the independent evolution of each genome leading to gain/loss of these MGEs. Conversely, ICEs from the SXT/R391 family showed a limited dissemination among *Shewanella* spp. These ICEs have been frequently found in the genus *Vibrio*, in some *Enterobacterales* (*Proteus*, *Providencia*, and *Klebsiella*) and in a few *Shewanella* spp. isolates, which reveals a narrow spectrum of hosts. Their modest distribution may be the result of several factors that affect the mating process, such as availability of surface receptors, environmental conditions that perturb mating-pair stabilization, the presence of exclusion mechanisms or defense systems in the recipient cell (Neil et al., 2021).

Furthermore, our work provides insightful data regarding plasmids circulating in *Shewanella* spp. We were able to identify replicases from broad host dissemination incompatibility groups (IncA/C, IncP, and IncX) frequently found in clinical isolates (Sekizuka et al., 2011; Carattoli et al., 2014; Jousset et al., 2018), as well as unique replicases in this genus, reflecting an unexpected diversity. In addition, several isolates from different lineages harbored more than one plasmid. The ability of these elements found in isolates from various sources (clinical or environmental) to replicate and coexist in different *Shewanella* spp. unfolds new scenarios involving HGT that could lead to a serious problem in the near future. Accordingly, recent reports showed that *Shewanella* spp. can have ARG-bearing plasmids, which share the same backbone as those found in common bacterial pathogens, such as *Enterobacterales*, from which they most likely were acquired (Sekizuka et al., 2011; Carattoli, 2013; Jousset et al., 2018).

Increase of ARGs in *Shewanella* spp. indicates that this genus is evolving steadily toward extensively drug resistance (XDR) phenotypes (Ohama et al., 2021). In this regard, several MDR and XDR isolates from our dataset were recovered from clinical samples or hospital environments. Notwithstanding, strain *Shewanella* sp. ALD9 had an MDR phenotype and it was isolated from a marine environment with limited human impact. The fact that this MDR isolate can be recovered from a niche where antibiotic pressure is probably slim, reflects that the ARG exchange can occur in their natural habitats and allows us to forecast its impact on the emergence of XDR *Shewanella* spp. in clinical settings that will lead to infections difficult to treat.

Evolution toward XDR in Gram-negative bacteria is commonly related to mobile integrons (Centrón and Roy, 2002; Hall, 2012). The link between class 1 integrons and ecosystems with high anthropic impact has been previously reported, as well as their contribution to the spread of ARGs (Chamosa et al., 2017). These integrons were also found in *Shewanella* spp. isolated from a bovine fecal sample (Barlow et al., 2004). Similarly, *S. baltica* CW2 carrying a class 1 integron was isolated from the gut of a lake trout (Castillo et al., 2018). The symbiotic relationship between some *Shewanella* species with marine animals provides an alternative environment where conditions may promote the acquisition of ARGs leading to an increase of the resistance level in non-human niches by single HGT events that may involve plasmids circulating in clinical isolates harboring mobile integrons.

Regarding the ARGs detected in this genus, we found the non-mobile colistin resistance gene *mcr* in a few *Shewanella* species. Zhang et al. (2019b) proposed that these genes may have emerged from bacteria found in aquatic niches. Since a few genes were found in only five *Shewanella* spp. isolates, it is unlikely that this bacterium is its native host. Furthermore, we observed quinolone and carbapenem resistance genes that were ubiquitous within specific pathogenic lineages. The association of *bla*_OXA–48–like_ and *bla*_OXA–729_ (former *bla*_OXA–55_) genes with *S. xiamenensis* and *S. algae*, respectively, has been previously reported (Tacão et al., 2018; Ohama et al., 2021). Here we propose a possible third link between *bla*_OXA–900_ and the *S. putrefaciens* lineage. Interestingly all these species belonged to the respective pathogenic lineages CG2 and CG1, which suggest that either they may have been acquired before speciation or that these genes were transferred by MGEs capable of inserting at specific loci in the chromosome. Further studies focused on the genetic surroundings of each of these genes employing a larger number of genomes may provide new insights into their ubiquitous nature. On the other hand, we found *qnrA* variants in *Shewanella* spp. from CG1, which is consistent with previous reports (Araújo et al., 2021; Huang et al., 2022). The fact that some *Shewanella* spp. encoding ubiquitous ARGs exposes a troublesome scenario where an isolate may be able not only to acquire MGEs carrying several ARGs, but also to become resistant to a broader spectrum of antibiotics by introducing point mutation in key genes. In this regard, enrichment analysis also showed the overrepresentation of GO terms related to ARGs *ampC*, *emrE*, *folM*, *folX*, *nagA*, *nfnB*, and *rfbC*.

Although *Shewanella* spp. can acquire ARGs-bearing MGEs (Parmeciano Di Noto et al., 2016; Jousset et al., 2018), *S. algae*, the most frequently reported species that causes infections in humans, showed the least diversity and frequency of plasmids and very few ARGs, which is consistent with previous reports (Huang et al., 2022). Conversely, most MDR and XDR isolates were found in the monophyletic group CG2. This cluster along with lineages *S. xiamenensis*, *S. baltica*, and *S. putrefaciens* had a higher rate of plasmids and ISs in their genomes, showing remarkable plasticity. The unique behavior of each lineage may be due to intrinsic features of the host that prevent plasmid invasion or maintenance, such as entry exclusion, restriction/modification and CRISPR-Cas systems (Hall, 2012). The formers are capable of hindering the invasion of plasmids and other MGEs, such as phages or ICEs, into a new host (Koonin and Makarova, 2009). Nevertheless, we found that isolates carrying these systems can also harbor different MGEs. Although a negative correlation among these elements has been previously reported for other bacteria; the opposite association has also been observed (Gophna et al., 2015; O’Meara and Nunney, 2019), which suggests that there are other factors that may govern this interaction. Accordingly, the sole presence of a CRISPR-Cas system in a genome does not denote activity. The type I-E from *Escherichia coli* is known for being tightly regulated and it is possible to turn it on under controlled laboratory conditions (Pul et al., 2010). Activity of CRISPR-Cas systems in *Shewanella* spp. may also be inhibited during DNA invasion by MGEs, and particular environmental conditions might be necessary to activate them. In the appropriate conditions, these systems may be fully functional and capable of acquiring immunity against new MGEs, evidenced by the presence of arrays with >40 CRISPRs. On the other hand, CRISPR-Cas systems may have been acquired by HGT events, and therefore its acquisition may have occurred after the invasion of an MGE. Last, it is known that MGEs may encode proteins, known as anti-CRISPRs, that overcome CRISPR-Cas machinery and evade host immunity (Stanley and Maxwell, 2018). Of note, Westra and Levin (2020) analyzed the interaction between phages and plasmids and proposed that it is dependent not only on MGE abilities but also on the strong complexity of each ecosystem. Further studies are necessary to unravel the interplay between these defense systems and MGE dissemination in a unique bacterium such as *Shewanella*.

A major question that requires further analysis is the identification of virulence genes that may be involved in *Shewanella* spp. pathogenesis. Our work led to the identification of various virulence genes in many species using two different approaches, comparative sequence analysis and GSEA. The implementation of GSEA allowed us to identify additional functional GO terms enriched in lineages comprising clinical samples that may encode certain molecular functions or biological processes contributing to their virulence. Among the virulence determinants detected or overrepresented in CG genomes, we found several genes related to adherence and colonization of different hosts (*wecE* and *ycdX*), toxicity (e.g., *toxA, cysC, plcN*, and *cylR*), secretion (T2SS, T6SS, and T3SS), swarming and swimming motility (e.g., *acf, che, fla, fle, fli, flg, ycdX*, and *yeaJ*), iron metabolism (e.g., *acs, pch, ccm, sit, ftnA*, *hmuR*, and *tuf*), among others. Interestingly, the T6SSs were found in all *S. algae* strains while gene *vgrG1* was enriched in CG1. Linares et al. (2016) suggested that these systems probably participate during *Shewanella*’s infection process. In order to confirm whether they are key pathogenicity factors in *S. algae*, further studies should be conducted. In addition, we found several other virulence genes encoded in this species, which were consistent with previous results (Wang et al., 2020; Zago et al., 2020). We also identified other potential candidate genes overrepresented in CG genomes, such as *speCEG* (spermidine synthesis), *potE* (putrescine transport) (Fang et al., 2017; Guerra et al., 2018), *cobS* (cobalamin biosynthesis) or genes related to oxidative stress response, specifically the peroxidase encoding gen *katG*. KatG has been previously reported as an important factor that may generate a stronger response during oxidative stress and increase survival during infection (Jiang et al., 2014). Many of these predicted virulence factors may have as main purpose to facilitate long-term interactions with other organisms or to collaborate with *Shewanella*’s survival and adaptation to different habitats (i.e., in aquatic niches or within a host). In this regard, metagenomic studies have shown the relevant abundance of members of the *Shewanella* genus in fish gut and invertebrates, which may vary under different conditions (Parris et al., 2016; Tepaamorndech et al., 2020; Jia et al., 2022; Johny et al., 2022). Moreover, these bacteria were also detected in the gastric and colorectal mucosal microbiomes of healthy and sick patients (Angelakis et al., 2016; Yang et al., 2022), showing an unexpected incidence. The true role of these factors during *Shewanella*’s colonization or infection processes in different hosts is still unknown.

The in-depth study of GSEA results also allowed us to identify an overrepresentation of certain paralogs genes in isolates from the CGs, suggesting that they may be involved in *Shewanella* spp. pathogenicity, i.e., *katG*, *cysQ*, *hemB*, *kefB*, and *modA* genes. *katG* paralogs are unevenly distributed between CGs and NCGs. Our results showed that an early duplication followed by a sequence divergence process may have occurred in the environment in both CGs. These genomes tend to maintain both paralogs, which might result in better fitted bacteria capable of thriving in clinical settings or during host infection/colonization. On the other hand, variants for genes *cysQ*, *hemB*, *kefB*, and *modA* showed lower identity values and they were mostly present in isolates from the CG. Each of these genes has the potential to contribute to *Shewanella* spp. virulence at different levels during a colonization or infection process, either adapting the environment to its own benefit or to increase its chances of survival against the host immune system. The differential retention of paralogs and its effects on the virulence of *Shewanella* spp. is something that deserves further investigation.

*Shewanella* spp. may cause infectious diseases in humans and in aquatic animals as well as to thrive as symbiont or in environmental niches. In the context of the One Health strategy, the exchange and evolution of genetic elements, such as MGEs or other GIs, seem to be contributing to the emergence of more virulent and resistant species and will affect their treatment with first line antibiotics in the near future. Our data allow us to evidence the ongoing transformation of the genus *Shewanella* into a worrisome pathogen with profound consequences for human and animal health. In this regard, we advocate that a more active role in the correct identification of this pathogen may reduce its potential hazard, thus improvements on the molecular identification of known and potential pathogenic lineages described in this study are necessary. In addition, since some virulent lineages, such as *S. algae* and *S. xiamenensis*, have ubiquitous resistance genes in their genomes, we propose their use as biomarkers.

Based on our results, we can attest that the evolution toward pathogenicity in this genus is a complex process that probably occurs idiosyncratically in each lineage. This process may involve long-term evolution of multiple genes, i.e., point mutations and duplications, in parallel with the continuous genetic exchange among bacteria. Further studies on *Shewanella* spp. virulence will provide an insight into the colonization and/or infection processes.

## Data availability statement

The authors confirm all supporting data, code, and protocols have been provided within the article or through Supplementary material and they can be found in the following links https://github.com/LBC-Iriarte/Shewanella_genomics and https://figshare.com/authors/QuirogaLab_IMPaM_UBA-CONICET_/14600396. All genome sequences (144) are listed in Supplementary Table 1, and GFF, FAA, and FNA files of the genomes are available at https://figshare.com/authors/QuirogaLab_IMPaM_UBA-CONICET_/14600396.

## Author contributions

AI and CQ: conceptualization, resources, and supervision. GC, GT, TA, AI, and CQ: data curation and visualization. GC, GT, TA, GP, MR, DC, AI, and CQ: formal analysis and writing—review and editing. CQ: funding acquisition. GC, GT, AI, and CQ: investigation, methodology, software, and writing—original draft. All authors contributed to the article and approved the submitted version.
