# Supplementary material for: Comparative genome analysis of the genus Shewanella unravels the association of key genetic traits with known and potential pathogenic lineages
Source: Front Microbiol. 2023 Feb 28;14:1124225. doi: 10.3389/fmicb.2023.1124225 (PMC10011109; doi:10.3389/fmicb.2023.1124225)
Supplement: Supplementary file 1 [file Presentation_1.pdf]

Supplementary Material - Figures

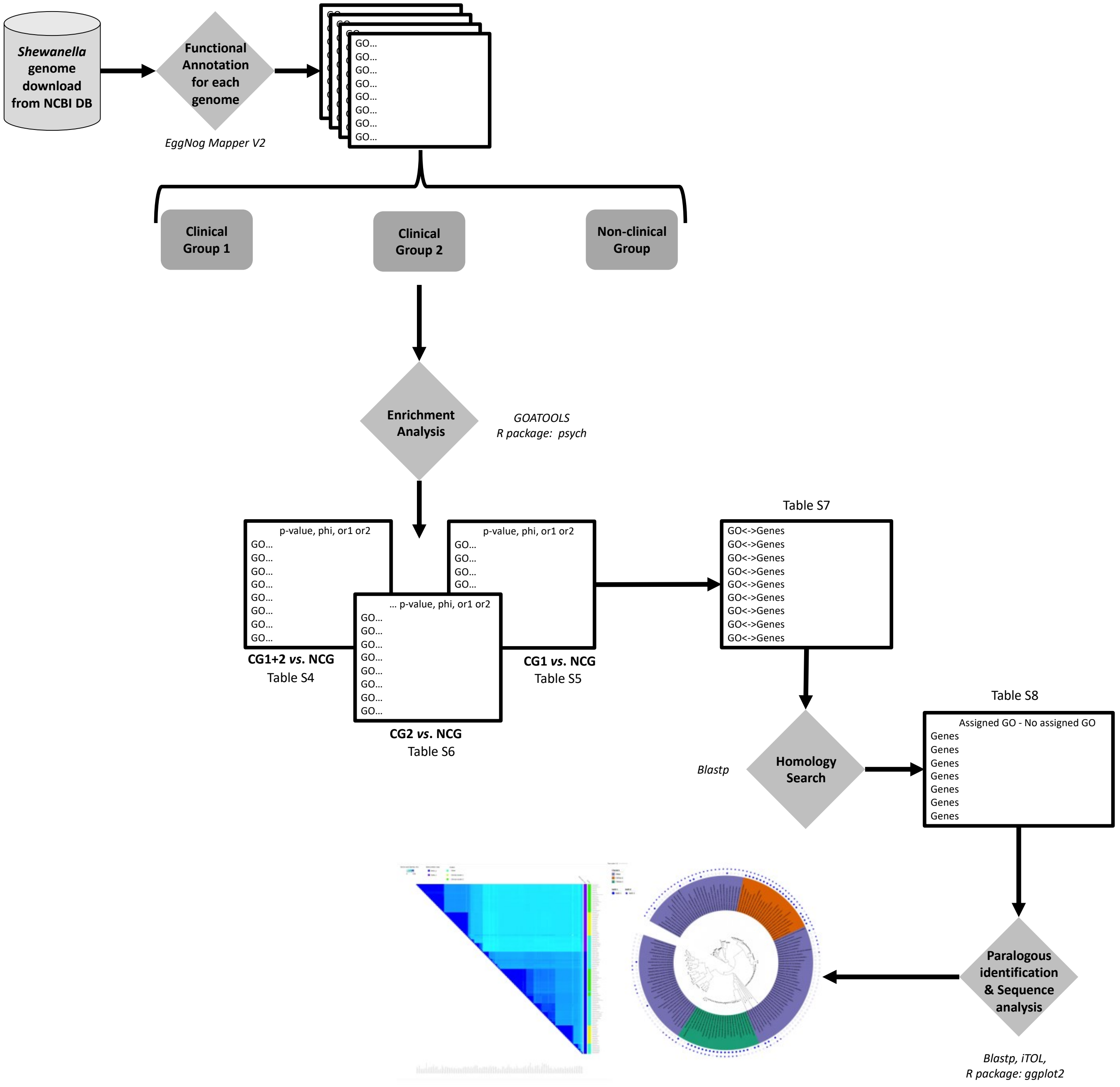

Figure S1 - Workflow for GO term enrichment analysis implemented in the present study.

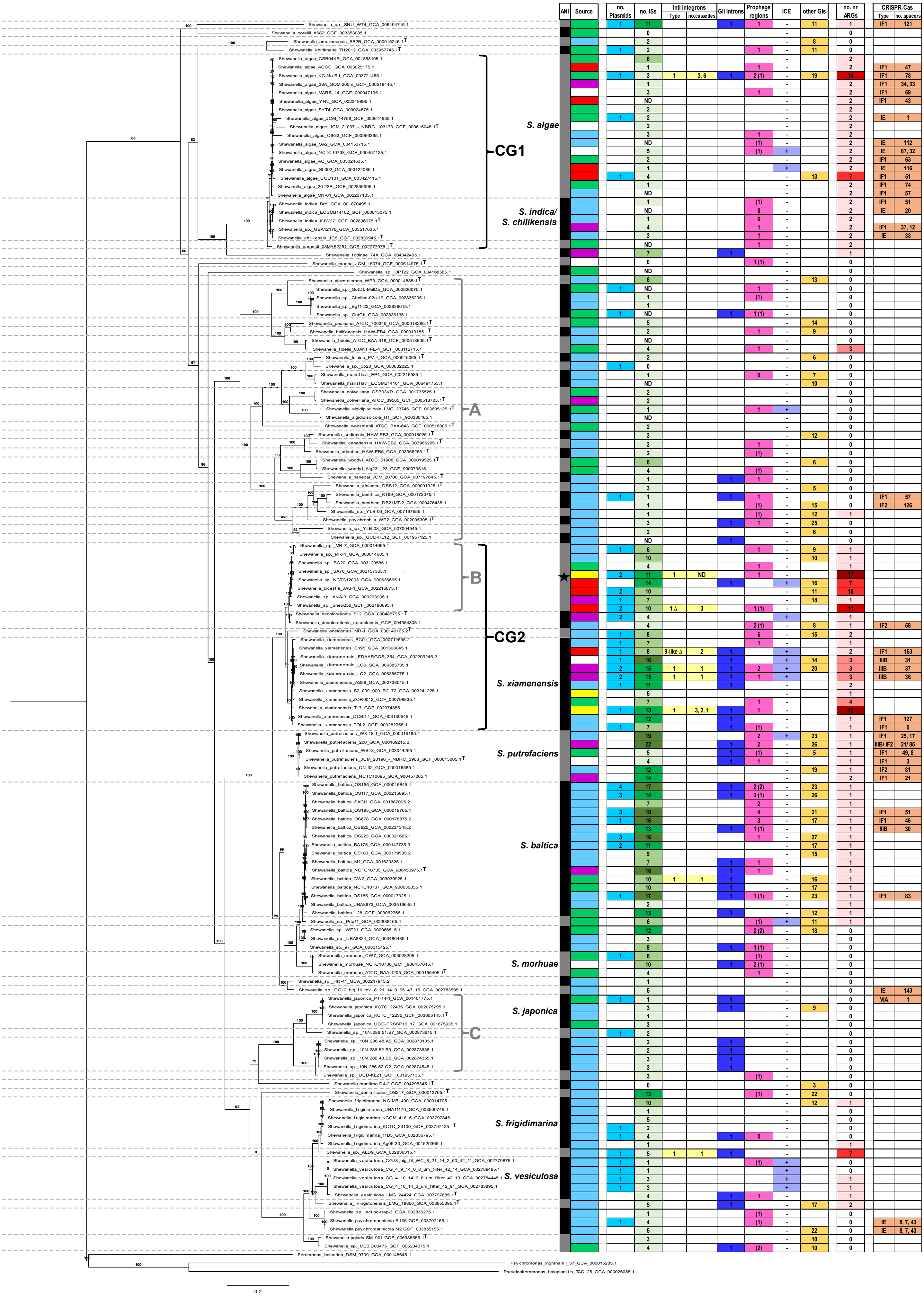

Figure S2 - Expanded phylogenomic summary tree. Dotted horizontal lines (- - -) separate monophyletic groups; numbers above branches indicate bootstraps support; Alteromonadales genomes were used as outgroups. ANI values are depicted with grey and black boxes. Black star indicates species with marginal ANI values. Letters A, B and C indicate mono- and polyphyletic lineages; CG1 (Clinical Group 1) and CG2 (Clinical Group 2) represent monophyletic groups with 100% statistical node support containing clinical isolates; T superscripts indicate type-strain isolates. Isolate sources are depicted with blue (aquatic niches/ sediments); green (animal-host) red (clinical); yellow (hospital environment); and violet (other impacted niches) colors. Each MGE and CRISPR-cas were identified in their respective columns with a specific color. Detailed information of each genetic feature can be found in the corresponding Supplementary Table.

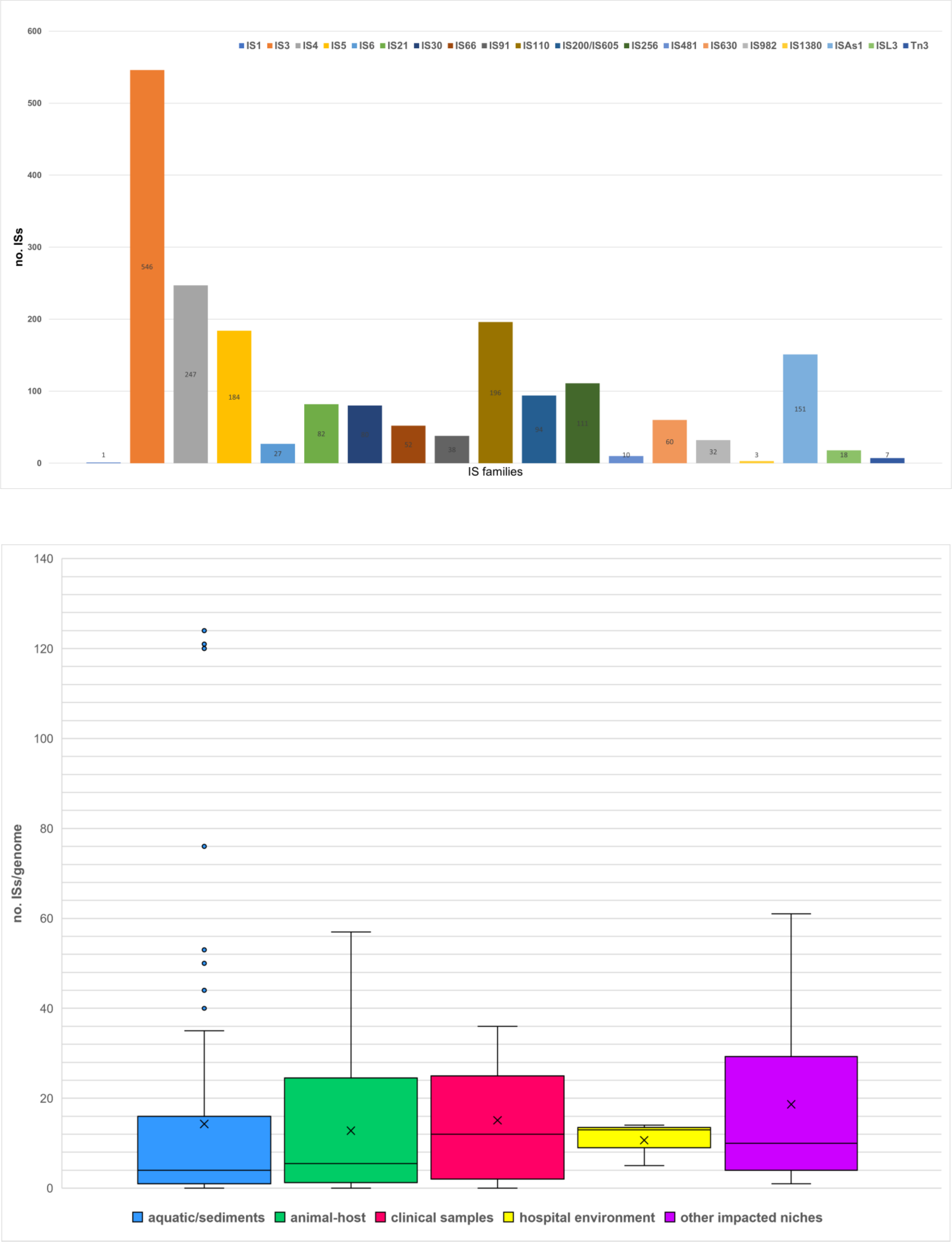

Figure S3. A. ISs in *Shewanella* spp. genomes per IS family. Columns identify the different IS families. Numbers inside each column indicate the total number of ISs (including redundancies) belonging to each IS family found in the pangenome of the genus *Shewanella* (Table S10). **B. ISs in *Shewanella* spp. genomes in different niches.** Boxplot displaying the number of ISs per genome in the different niches. Outliers are depicted as dots.

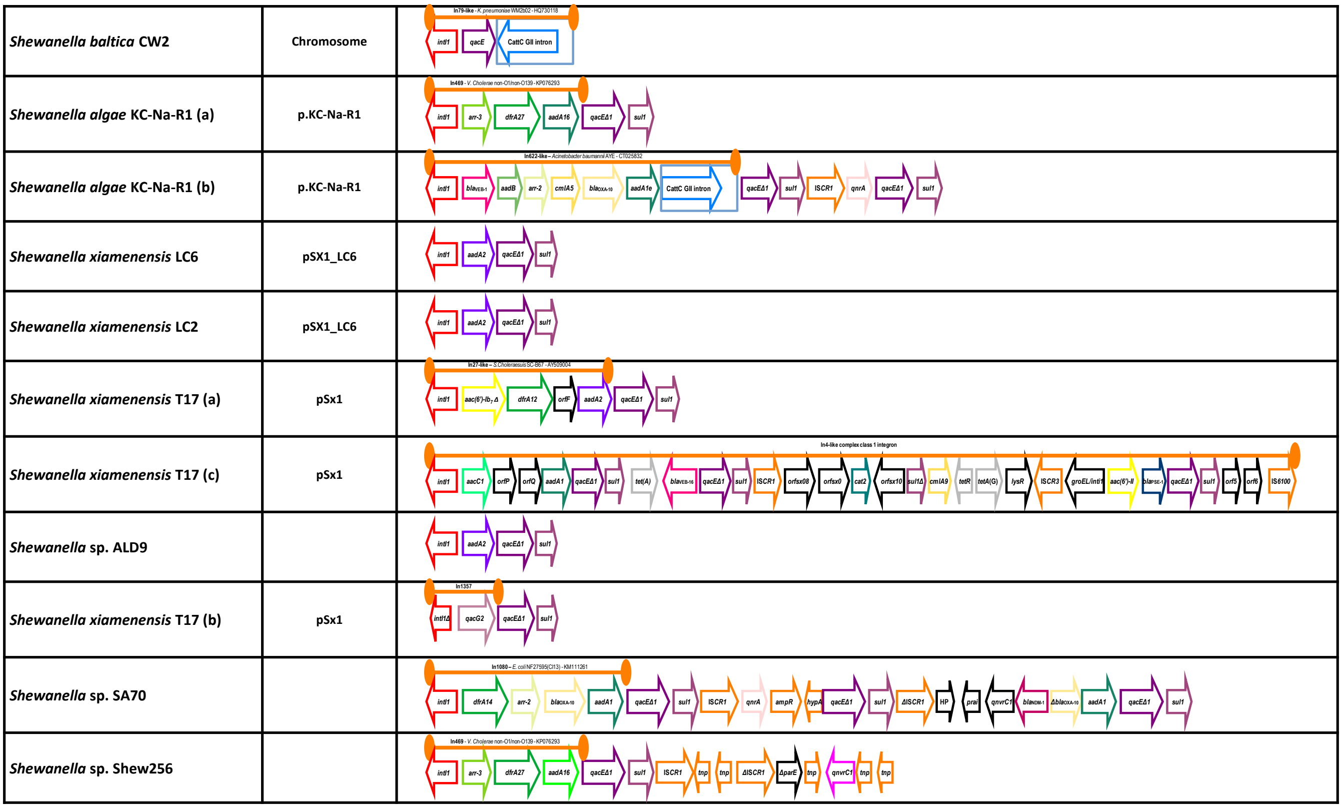

Figure S4. Class 1 Integrons found in *Shewanella* spp. genomes. Each gene is represented with horizontal arrows and their length is according to the size of the gene. *intI1* genes are shown in red, orange horizontal lines represent known class 1 integrons; MGEs are depicted with orange arrows and GII introns with blue rectangles and arrows.

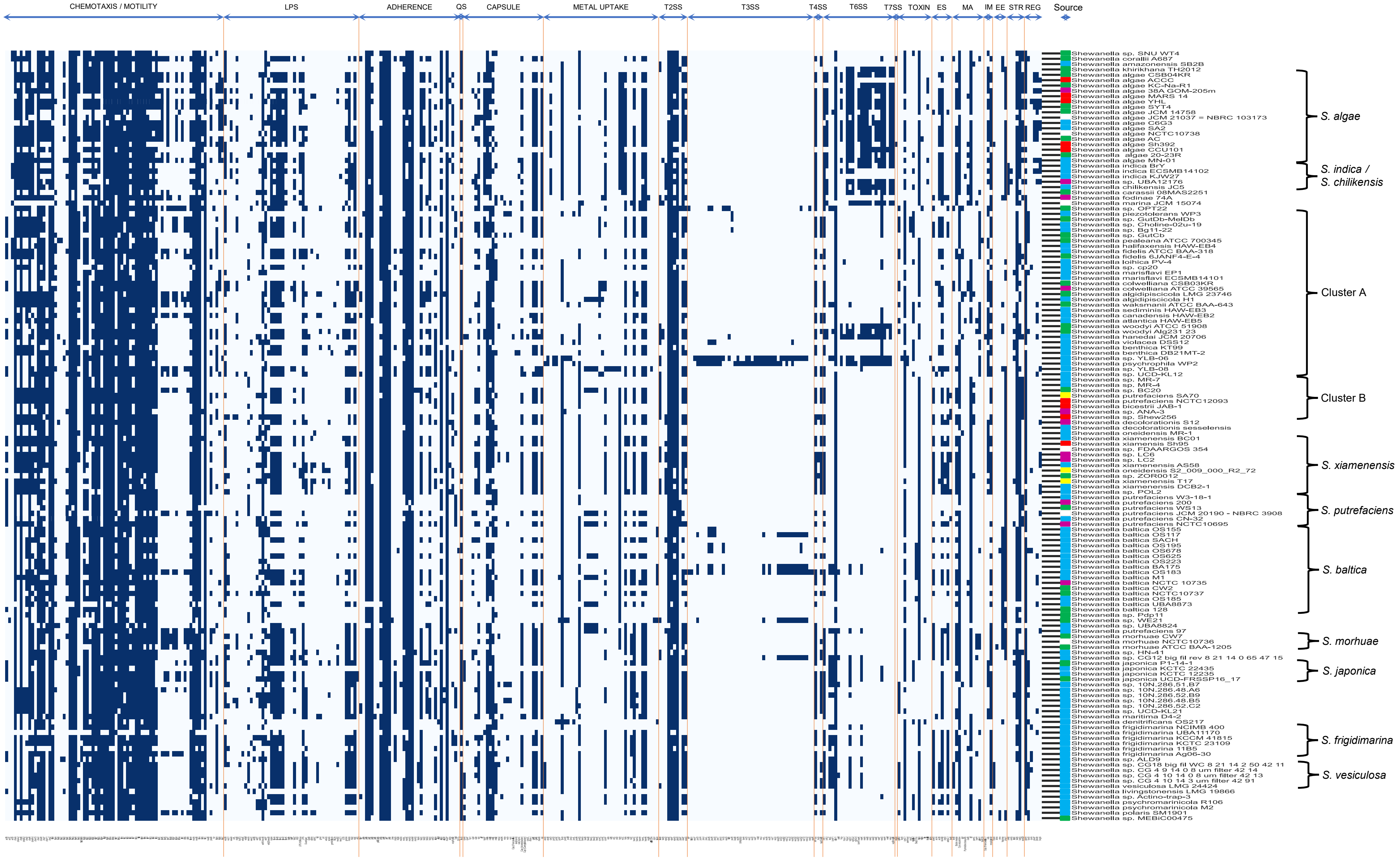

Figure S5 - Heat-map of virulence factor genes found in *Shewanella* spp. genomes. *Shewanella* spp.genomes (n=144) were sorted according to the phylogenetic tree (y-axis) and virulence factor genes (x-axis), Genes and genomes were depicted based on the organization showed in the virulome supplementary table (Table S14) and phylogenomic tree from Figure S2. Each major category was separated with vertical lines.

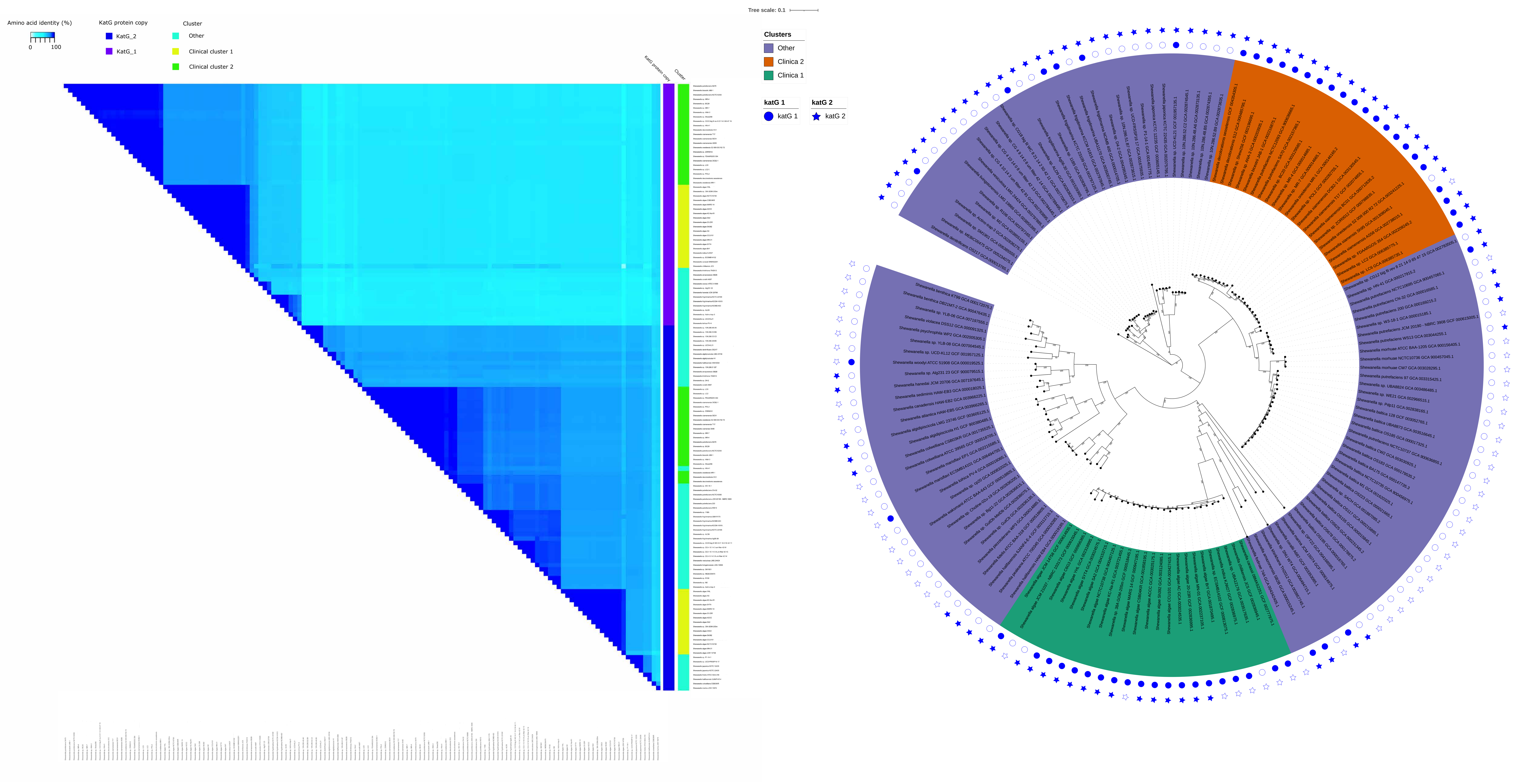

Figure S6.Sequence analysis of KarG protein among *Shewanella spp. genomes.* A. The heatmap indicates the variability of amino acid changes of KatG copies between Shewanella spp. genomes. On the right, the first column showa the protein variants (KatG_1: red; KatG_2: yellow), and the second indicates the cluster it belongs to (Clinical1: green; Clinical 2: orange; Other: violet). B. Distribution of different KatG protein variants in the Shewanella spp. phylogeny. The hallmarks indicate the different clusters, and the circles and stars represent variants KatG_1 and KatG_2, respectively. If the symbol is full, it shows its presence, and if the symbol is hollow, it shows absence.

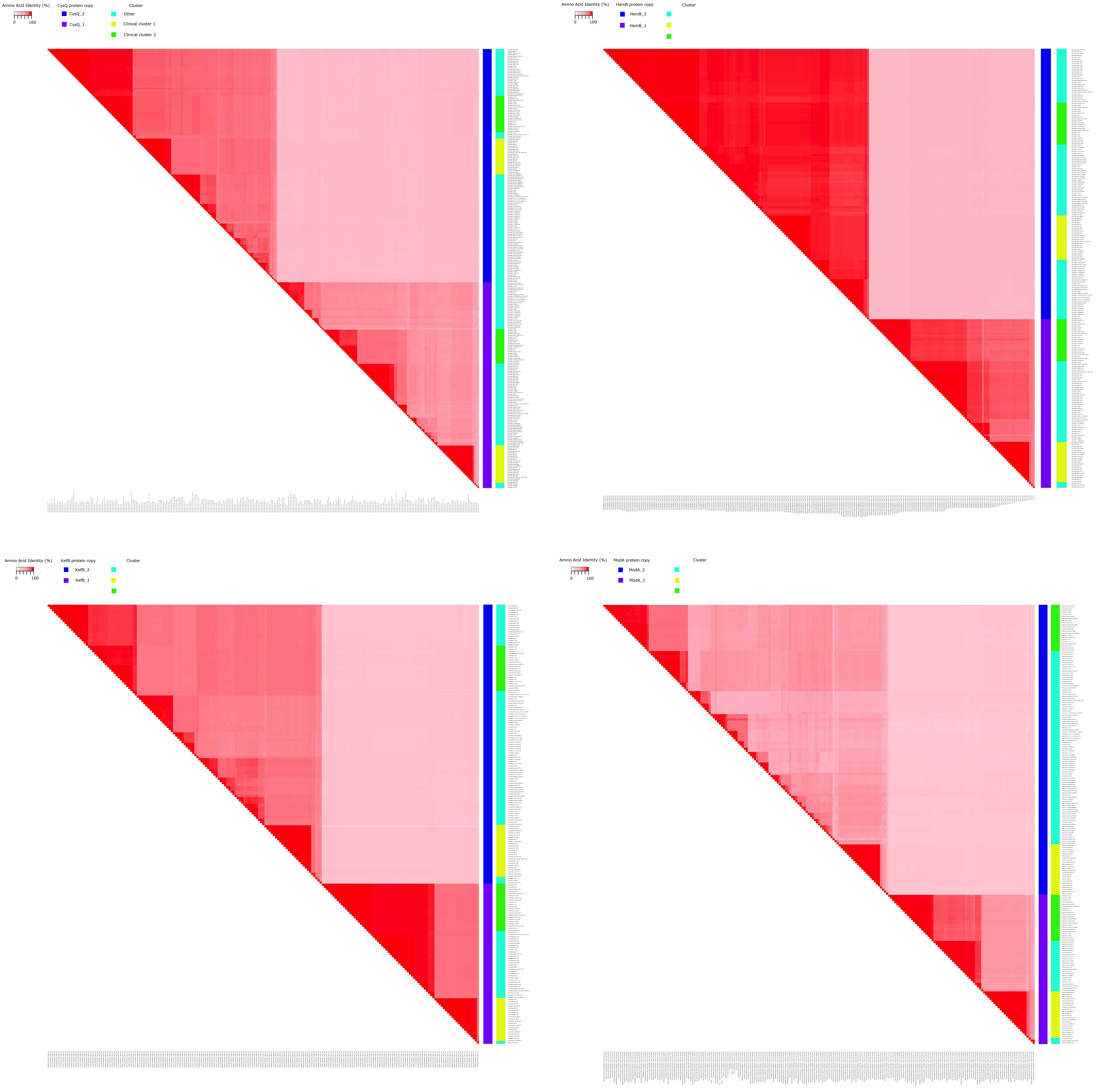

Figure S7.Sequence analysis of CysQ, HemB, KefB and ModA proteins among *Shewanella spp. genomes.* Each heatmap indicates the protein identity between all copies. Top left: CysQ; top right: HemB; bottom right: ModA. First columns show the protein variant (blue and violet columns); second columns represent they cluster each variant belong to (Clinical 1: yellow; Clinical 2: green; Other: cyan).
